# How are academic achievement and inhibitory control associated with physical fitness, soil-transmitted helminth infections, food insecurity and stunting among South African primary schoolchildren?

**DOI:** 10.1186/s12889-021-10779-9

**Published:** 2021-05-03

**Authors:** Markus Gerber, Christin Lang, Johanna Beckmann, Rosa du Randt, Stefanie Gall, Harald Seelig, Kurt Z. Long, Sebastian Ludyga, Ivan Müller, Madeleine Nienaber, Siphesihle Nqweniso, Uwe Pühse, Peter Steinmann, Jürg Utzinger, Cheryl Walter

**Affiliations:** 1 Department of Sport, Exercise and Health, University of Basel, St. Jakob-Turm, Birsstrasse 320B, 4052 Basel, Switzerland; 2 Nelson Mandela University, Port Elizabeth, South Africa; 3 University of Basel, Basel, Switzerland; 4 Swiss Tropical and Public Health Institute, Basel, Switzerland

**Keywords:** Executive functions, Fitness, Food insecurity, Soil-transmitted helminths, Stunting

## Abstract

**Background:**

Cardiovascular fitness has been associated with both executive function and academic achievement in multiple cohort studies including children and adolescents. However, research is scarce among children from low- and middle-income countries. Hence, this paper focuses on South African primary schoolchildren living in marginalized areas and examines if academic achievement and inhibitory control can be explained by children’s age, socioeconomic status, soil-transmitted helminth infections, food insecurity, stunting, grip strength, and cardiorespiratory fitness.

**Methods:**

The sample of this cross-sectional study consisted of 1277 children (48% girls, mean age: 8.3 years). Data were assessed via questionnaires, stool samples, anthropometric measurements, 20 m shuttle run test, grip strength test, Flanker task, and school grades. Data were analysed with mixed linear regression models with random intercepts for school classes, separately for boys and girls.

**Results:**

Higher socioeconomic status was most closely associated with academic achievement among boys (*p* < 0.05), whereas higher levels of cardiorespiratory fitness and not being stunted explained most variance in academic achievement in girls (*p* < 0.05). Higher age turned out to be associated with better performance in the Flanker task (*p* < 0.01). Additionally, in boys, higher grip strength was associated with better information processing and inhibitory control of attention (*p* < 0.01), whereas in girls, higher cardiorespiratory fitness levels were positively associated with these cognitive abilities (*p* < 0.05).

**Conclusions:**

Academic performance has been shown to be compromised in schoolchildren living in marginalised areas, compared to schoolchildren in less disadvantaged parts of South Africa. The present study suggests that cardiorespiratory fitness and grip strength are two potentially modifiable factors that are associated with children’s academic achievement and cognitive performance, and that should be targeted in future school-based interventions.

## Introduction

Among the different cognitive domains, executive function has been identified as an important (positive) predictor of fluid and crystallised intelligence [1], school readiness [2], academic achievement [3] and mental wellbeing [4]. While most of the evidence on the relationship between executive function and academic performance comes from high-income countries, a recent study among West African preschool-aged children corroborates that executive function predicts children’s literacy and numeracy skills also in low-resource settings [5]. Researchers recently found that in South African children higher socioeconomic status is associated with better executive function performance [6], and that higher gross motor skills were positively associated with some (but not all) executive function components [7]. Executive function includes and is defined as a multitude of different cognitive processes that are coordinated for goal-directed behaviour and problem-solving [8]. This domain can be subdivide into (1) inhibitory control (suppression of irrelevant stimuli or overriding a prepotent response), (2) working memory (holding information in mind and manipulating information) and (3) cognitive flexibility (ability to shift between mental sets) [9]. In a recent conceptualization of executive function, inhibitory control has been suggested to be common to all of its subcomponents.

Beyond executive functions, children’s academic achievement is influenced by a multitude of factors including educational opportunities, socioeconomic status (SES), health and nutritional status, as well as the family environment. Again, most of the current empirical evidence is based on children from high-income countries [10], although similar associations have been described in low- and middle-income countries (LMICs) [11, 12]. However, children living in socioeconomically deprived environments are facing additional challenges [13]. For instance, if most of the available resources of a family are used to cover basic needs (e.g., food and housing), it is often difficult for parents to assure a stimulating learning environment and to support their children in academic matters. As shown by Liddell and Rea [14], only 39% of all children in rural South Africa progressed through primary school without disruption, whereas 36% had left their original school, and 25% have been retained at least once. Venter and Bham [15] further highlight that school failure among grade 1 students and subsequent grades dropout is a particularly serious concern among students from marginalized, non-English speaking communities, compared to children living in more privileged settings. Moreover, living in poverty increases the likelihood of chronic malnutrition among children [16], which can manifest in stunted growth. Stunting, in turn, has been associated with poor cognitive function, low intelligence quotient (IQ), and academic performance [11]. Accordingly, stunted children are at increased risk of poor scholastic success already in grade 1, which may have severe negative impact on their future school performance and their subsequent well-being.

Furthermore, meta-analytical findings suggest that physical activity interventions elicit small, but significant benefits for cognitive function [17, 18] and academic achievement [19, 20]. These benefits may partly depend on the influence of physical activity on different facets of physical fitness, given that high cardiorespiratory fitness (CRF) in particular has been related to both high executive function and academic achievement in large cohorts of children and adolescents [21, 22].

Despite the solid evidence base, research on the interplay between physical activity, fitness, cognitive function and academic achievement is sparse among children from LMICs. In a study with 835 learners aged 8–12 years from eight primary schools in marginalized neighbourhoods in the Eastern Cape Province of South Africa, Gall et al. [23] found that low selective attention was associated with soil-transmitted helminth (STH) infections and relatively lower physical fitness, whereas higher academic achievement was observed in children without STH and higher physical fitness levels. Similar findings are reported in a recent study among 172 primary school girls (6–13 years old) from the North West Province of South Africa [24], where CRF was found to mitigate the negative consequences of unfavourable body composition on academic achievement. Haywood and Pienaar [24] therefore concluded that “physical fitness should be used strategically in preventive intervention programs necessary to enhance cognitive functioning, academic performance and brain health among overweight children” (p. 1).

The purpose of the present study was to assess whether and to what degree academic achievement (school grades) and inhibitory control in primary school children (grades 1–4) can be explained by their age, SES, STH infections, food insecurity, stunting, grip strength, and CRF. We expected that children with higher SES, who are not stunted, who are not infected with STH and with better physical fitness will show higher academic achievement and higher inhibitory control.

## Methods

### Study design

The present paper is based on cross-sectional baseline data of the *KaziAfya* cluster randomized controlled trial (registration date: August 9, 2018, with ISRCTN; https://doi.org/10.1186/ISRCTN29534081) on the effectiveness of increased physical activity and multi-micronutrient supplementation. The baseline data assessment took place from January to April 2019. Based on an a-priori power analysis [25], we intended to recruit 1320 South African primary schoolchildren from grades 1–4 (aged approx. 6–12 years).

### Participants and procedures

Four quintile 3 schools located in peri-urban areas in Port Elizabeth (South Africa) were recruited. South African public schools are classified into five groups, with quintile 1 standing for the poorest and quintile 5 for the least poor school environment. School authorities were first contacted before contacting schools through the school principals who were informed about the objectives, procedures and potential risks/benefits of the study.

Written informed consent was obtained from the parents/guardians of the children before the start of the baseline data assessment. After having signed written informed consent, the parents/guardians were interviewed regarding the families’ SES and dietary intake. Additionally, all children participating in the study provided oral assent before the start of the study.

Schools were eligible for the study if they were public quintile 3 schools located in marginalized areas, if facilities were available for the implementation of physical education lessons and if they did not engage in any other research project, clinical trial, or were part of governmental nutrition interventions. Children had to meet the following inclusion criteria: (i) attend grade 1–4; (ii) not be older than 12 years; (iii) have written informed consent from their parents/guardians; (iv) not participate in other research projects, clinical trials, or food/nutritional programmes; and (vi) not suffer from clinical conditions that prevent participation in physical activity, as determined by qualified medical personnel.

### Ethical considerations

Ethical approval was obtained from the ‘Ethikkommission Nordwest- und Zentralschweiz’ in Switzerland (EKNZ; reference number: Req-2018-00608). The intervention study was registered in the ISRCTN registry (https://www.isrctn.com/ISRCTN29534081). Approval has also been obtained in South Africa from the research ethics committee (REC-H) of the Nelson Mandela University in Port Elizabeth (reference number: H18-HEA-HMS-006) and the Eastern Cape Department of Education of the Eastern Cape Province. Children excluded after diagnosis of a relevant medical conditions and/or children suffering from malnourishment (as diagnosed by a nurse, following national guidelines) were referred to the nearest local clinic.

### Data assessment

Data collection took place over several days at the schools between January and April 2019. Data assessment procedures used here were based on a set of standardised, validated and quality-controlled standard operating procedures [25]. Hand filled data sheets were double-entered and validated using EpiData (version 3.1, EpiData Association; Odense, Denmark) and merged into a single database.

#### Anthropometric measurements

Body weight was assessed via an electronic scale (Tanita MC-580, Tanita Corp.; Tokyo, Japan). The participants were asked to fast for 3 h prior to the data assessment, and to void their bladder immediately before the assessment. Body weight was measured with shoes off to the nearest 0.1 kg. To assess the body height, each child stood against a stadiometer with the back erect and shoulders relaxed (and with shoes off). Body height was measured to the nearest 0.1 cm. Sex-specific height-for-age z scores were computed from the WHO growth reference data [26]. In addition, children were classified as stunted if they had a height-for-age score below two standard deviations of the WHO Child Growth Standards median [27].

#### Socioeconomic status and food insecurity

A parental survey was used to assess children’s family socioeconomic status (SES) and food insecurity. To estimate SES, parents/guardians answered nine items, covering household-level living standards, such as infrastructure and housing characteristics (house type, number of bedrooms and people per household, type of toilet, and source of water, and electricity) and questions related to the ownership of three durable assets (presence of a working refrigerator, washing machine, and car). The dichotomized items (0 = poor quality, not available; 1 = high quality, available) were summed up to build an overall SES index, with higher scores reflecting higher SES. The validity of similar measures has been established in previous research [28].

We used three items from the Household Food Insecurity Access Scale [29] to assess food insecurity (In the past 30 days, did (a) all of your household members have access to enough food on every day? (b) you or any household member go to sleep at night hungry because there was not enough food? (c) you or any household member go a whole day and night without eating anything at all because there was not enough food?). This instrument has been validated in industrialized countries and LMICs. For example, Knueppel et al. [30] showed satisfactory validity and reliability among poor households in rural Tanzania. In line with the definition of USAID [31], food insecurity was defined as a situation in which not all people of a family have access to sufficient food to meet their dietary needs for a productive and healthy life. Responses of the dichotomized items (enough access = 0, not enough access = 1) were summed up to generate an overall food insecurity index, ranging from 0 (food secure/not hungry) to 3 (food insecure/hungry). The internal consistency of the three items in the present sample was satisfactory (Cronbach’s alpha = 0.74).

#### Soil-transmitted helminth infections

Pre-labelled stool sample containers were distributed to each class. These containers were taken home by the study participants and returned to the research assistant in the morning of the following day. All stool samples were processed on the day of collection in the laboratory at the study site. The Kato-Katz technique [32] was used to detect parasitic infections, including the common STH (*Ascaris lumbricoides*, *Trichuris trichiura*, and hookworms). Stool samples (at least 10–15 g) were first visually examined for the presence of blood, mucus and diarrhoea. Then, duplicate 41.7 mg Kato-Katz thick smears were prepared from each stool sample. Moreover, a random sample of 10% of the Kato-Katz slides were re-examined by a senior technician for quality control. In case of discordant results, the slides were read a third time, and the results discussed among the technicians until a common consensus was reached [33].

#### Physical fitness

We used the 20 m shuttle run test [34] to assess children’s CRF. Pre-recorded sound signals were played to the children, and they were allowed to do a trial run of 2 intervals (40 m) under the supervision of a research assistant. Once children were familiar with the test procedures, they ran back and forth on the 20 m flat course (marked with colour-coded cones) in groups of 10–15 children, following the pace of the sound signal. Starting with a running speed of 8.5 km/h, the frequency of the signal increased every minute by 0.5 km/h. When a child failed to follow the pace in two consecutive intervals, the last valid stage and corresponding speed was recorded. The speed and age of the participating child was to predict peak maximal oxygen uptake (VO_2_max) [34].

Upper body strength was determined with the grip strength test, with both right and left hand using the Saehan hydraulic hand dynamometer (MSD Europe BVBA; Tisselt, Belgium. Before the start of the test, the hand span (distance from the tip of the thumb to the tip of the little finger) of the child’s dominant hand was measured (to the nearest 0.5 cm), and the grip span on the dynamometer was adjusted accordingly [35]. The child held the dynamometer, while sitting in an upright position, shoulders adducted and neutrally rotated with the arm at right angles and the elbow at the side of the body. The forearm was in a neutral position with the thumb pointing upwards and the wrist between 0 and 30° of flexion and between 0 and 15° ulnar deviation. During this time, no other parts of the body touched the dynamometer, neither was the arm being tested squeezed against the body. Each child had six trials in total (with a 30 s rest in between) to grip the dynamometer as hard as possible with alternating hands. The results of each trial were assessed (to the nearest 1 kg) and the average was calculated to obtain an overall score.

#### Inhibitory control

Inhibitory control was measured with a computer-based version of the Flanker task [36] that was administered via E-Prime 2.0 Software (Psychology Software Tools, USA). This task serves as a standard test for assessing inhibitory control of attention and requires participants to respond to the direction of a centrally presented target stimulus, while flanking stimuli face in the same (congruent trials) or opposite direction (incongruent trials). The white fish (vertical visual angle: 1.8°; distance between fish: 1.4°) served as children-friendly visual stimuli and were presented against black background. Participants were asked to respond by pressing a button corresponding to the direction of the target stimulus. Prior to the testing, participants were instructed by the investigator and speed and accuracy were equally emphasized. Following two practice rounds with 60 trials in total, participants completed two blocks with 40 trials each. The test blocks were interspersed by a 30 s recovery period. The order of the trials was randomized and they appeared with equal probability. Visual stimuli were presented focally and the response window was set to 2500 ms. The inter-stimulus interval varied randomly between 1100 and 1500 ms to reduce a potential influence of guessing. Performance was assessed by calculating the mean accuracy as well as the mean reaction time (for correct responses only) separately for both trial types. Congruent trials provided a measure of information processing, whereas incongruent trials assessed inhibitory control of attention. In previous studies, the Flanker task proved to have high test-retest reliability [37] as well as adequate convergent and discriminant validity [38].

#### Academic achievement

End of year results of three subjects were used as an indicator of academic achievement: home language, mathematics and life skills. The South African school system uses a seven-point grading scale (from 1 [0–29%] to 7 [80–100%, with distinction]), with seven representing the highest possible grade. With a grade of 3 (≥40%), the students receive a “pass”.

### Statistical analyses

Normal distribution of the collected data was examined by applying the Kolmogorov-Smirnov and Shapiro-Wilk tests. Descriptive statistics are reported as M and SD or n and percentage (%), for the total sample, and separately for boys and girls. Differences between boys and girls in the study variables were tested with univariate analyses of variance (ANOVAs) or χ^2^-tests. Kolmogorov-Smirnov, Shapiro-Wilk tests, descriptive statistics, ANOVAs and χ^2^-tests were performed with SPSS Version 26 (IBM Corporation, Armonk NY, USA) for Mac.

A series of different mixed linear regression models with random intercepts for school classes were performed to examine the extent to which children’s age, SES, body mass index (BMI), food insecurity, stunting, STH infection status (not infected versus a single or multiple infection), grip strength, and CRF explained inhibitory control and academic achievement. After detection of considerable sex differences (see results section for more details), separate analyses were carried out for boys and girls. Mixed linear regression models were calculated with the Mplus software (version 7, Muthen & Muthen, 1998–2020) with a robust maximum likelihood estimator (MLR). These main analyses were based on the full sample, hereby handling missing data via full information maximum likelihood (FIML). To examine the prerequisites of FIML, we performed Little’s missing completely at random (MCAR) test, using SPSS. With regard to inhibitory control, the analyses were repeated, after exclusion of children who did not perform higher than chance (≤50%) in the Flanker task, to reduce the potential bias introduced by a failure to understand and follow task instructions. To interpret the findings of the mixed linear regression analyses, the following statistical coefficients were displayed: (a) Estimate (standardized Beta-weight), (b) standard error (S.E.) of the estimate, and (c) *p*-value. For all statistical analyses, the level of significance was set at *p* < 0.05.

## Results

### Sample characteristics, descriptive statistics, test of normality and inspection of missing data

From the 1369 children with informed consent, 65 dropped out before the baseline data assessment took place, most of them due to relocation or because they left or changed school. Of the remaining 1304 children, 27 were not considered in the data analysis because no information on sex was available. Accordingly, the final sample of the present paper consisted of 1277 children (664 boys, 613 girls), with a mean age of 8.3 years (SD = 1.4). Sample characteristics and descriptive statistics of all independent and dependent variables are presented in Table 1. With the exception of reaction time for congruent and incongruent stimuli, Kolmogorov-Smirnov and Shapiro-Wilk tests indicated that none of the dependent variables were normally distributed (*p* > 0.05). Hence, we used MLR in the mixed linear regression analyses to handle non-normal distribution of dependent variables. Table 1 also shows that the number of missing values differed considerable across study variables. Thus, whereas 1277 children had valid data for age, only 754 had valid data on SES. Little’s MCAR tests showed that data were missing completely at random, χ^2^(df = 1406) = 1434.5, *p* = 0.293, so FIML could be applied to impute missing data.

**Table 1:** Characteristics of the study population

		Total	Males	Females			
**Age and anthropometry**	***N***	***M*** **(*SD*)**	***M*** **(*SD*)**	***M*** **(*SD*)**	***F***	***p*-value**	**η**^**2**^
Age (years)	1277	8.3 (1.4)	8.4 (1.5)	8.2 (1.4)	6.2	0.013	0.005
Height (cm)	1240	124.7 (9.2)	125.1 (9.0)	124.3 (9.4)	2.9	0.087	0.002
Weight (kg)	1240	25.4 (6.9)	25.4 (6.6)	25.4 (7.2)	0.0	0.884	0.000
BMI (kg/m^2^)	1240	16.1 (2.6)	16.0 (2.4)	16.1 (2.8)	0.5	0.470	0.000
**Sociocultural characteristics**	***N***	***M*** **(*SD*)**	***M*** **(*SD*)**	***M*** **(*SD*)**	***F***	***p*-value**	**η**^**2**^
Socioeconomic status (SES)	740	3.0 (1.2)	3.0 (1.2)	3.0 (1.2)	0.4	0.539	0.001
Food insecurity*	940	1.3 (0.6)	1.3 (0.6)	1.2 (0.6)	2.6	0.111	0.003
**Helminth infections and stunting**	***N***	***n*** **(%)**	***n*** **(%)**	***n*** **(%)**	**χ**^**2**^	***p*-value**	
Infected	1245	89 (7.1)	50 (7.8)	39 (6.5)	0.8	0.383	
Stunted	1231	112 (9.1)	48 (7.6)	64 (10.7)	3.6	0.057	
**Physical fitness**	***N***	***M*** **(*SD*)**	***M*** **(*SD*)**	***M*** **(*SD*)**	***F***	***p*-value**	**η**^**2**^
Grip strength (in kg)	1277	10.3 (12.0)	11.8 (12.9)	9.7 (10.9)	2.5	0.114	0.002
Cardiorespiratory fitness (VO_2_max; in ml kg^−1^ min^−1^)	1277	43.9 (22.9)	44.1 (22.9)	43.7 (22.9)	0.1	0.761	0.000
**Academic achievement**	***N***	***M*** **(*SD*)**	***M*** **(*SD*)**	***M*** **(*SD*)**	***F***	***p*-value**	**η**^**2**^
End of year results	1045	4.6 (1.3)	4.4 (1.2)	4.8 (1.3)	35.9	0.000	0.033
Language	1045	4.5 (1.3)	4.3 (1.2)	4.8 (1.3)	36.3	0.000	0.034
Mathematics	1045	4.7 (1.3)	4.4 (1.2)	4.9 (1.4)	30.4	0.000	0.028
Life skills	1045	5.0 (1.0)	4.8 (1.0)	5.2 (1.0)	39.7	0.000	0.037
**Flanker task**	***N***	***M*** **(*SD*)**	***M*** **(*SD*)**	***M*** **(*SD*)**	***F***	***p*-value**	**η**^**2**^
Accuracy (congruent stimuli)	1223	92.8 (10.8)	92.6 (11.1)	92.9 (10.5)	0.2	0.651	0.000
Accuracy (incongruent stimuli)	1223	84.9 (19.4)	85.4 (18.2)	84.4 (20.6)	0.7	0.397	0.001
Reaction time (congruent stimuli)	1223	1187.9 (240.4)	1155.7 (243.5)	1222.2 (232.3)	23.9	0.000	0.019
Reaction time (incongruent stimuli)	1223	1273.6 (266.6)	1238.6 (262.5)	1310.9 (266.0)	22.7	0.000	0.018

Notes. *26 children (2.8%) had a score of 0 (fully food secure), 685 children (72.9%) had a score of 1, 172 children (18.3%) had a score of 2 and 57 children (6.1%) had a score of 3 (severe food insecurity)

### Differences between boys and girls

Table 1 shows that boys and girls differed in several study variables. More specifically, boys were taller, and had lower academic results. However, boys performed better in some indices of the Flanker task, achieving faster reaction times. No significant differences were found with regard to SES, food insecurity, STH infections, stunted growth, grip strength and CRF.

### Multivariate analyses to explain academic achievement

Table 2 shows that among boys, few factors were associated with their academic achievement. Higher SES was related to better end of the year results and grades in life skills and mathematics. Moreover, higher CRF was associated with better grades in mathematics, whereas stunted growth was related to poorer grades in the life skills subject.

**Table 2:** Multiple linear regression analyses to explain academic achievement in boys (*n* = 664) and girls (*n* = 613)

**End of the year results**	**Mixed multiple linear regression**					
	Boys			Girls		
Explanatory variables	Estimate	S.E.	*p*-value	Estimate	S.E.	*p*-value
Age	0.00	0.05	0.956	−0.07	0.08	0.382
BMI	0.04	0.06	0.533	0.06	0.06	0.290
Socioeconomic status	**0.14**	**0.007**	**0.043**	−0.06	0.06	0.326
Stunting (0 = not stunted, 1 = stunted)*	− 0.05	0.05	0.297	**− 0.11**	**0.05**	**0.038**
Food insecurity	0.06	0.07	0.324	−0.09	0.06	0.061
STH infection (0 = not infected, 1 = infected)*	0.01	0.04	0.687	−0.12	0.06	0.053
Grip strength	0.03	0.05	0.562	0.10	0.08	0.205
Cardiorespiratory fitness	0.08	0.05	0.101	**0.14**	**0.07**	**0.031**
**Language**	**Mixed multiple linear regression**					
	Boys			Girls		
Age	−0.05	0.06	0.398	−0.11	0.08	0.185
BMI	0.03	0.06	0.630	0.09	0.06	0.131
Socioeconomic status	0.12	0.07	0.065	−0.08	0.06	0.207
Stunting (0 = not stunted, 1 = stunted)*	−0.06	0.05	0.181	**−0.10**	**0.05**	**0.038**
Food insecurity	0.10	0.07	0.130	−0.09	0.06	0.129
STH infection (0 = not infected, 1 = infected)*	− 0.02	0.04	0.568	−0.11	0.06	0.065
Grip strength	0.02	0.05	0.747	0.10	0.08	0.176
Cardiorespiratory fitness	0.05	0.05	0.248	**0.14**	**0.06**	**0.028**
**Mathematics**	**Mixed multiple linear regression**					
	Boys			Girls		
Age	0.05	0.05	0.302	−0.03	0.08	0.683
BMI	0.04	0.06	0.462	0.03	0.06	0.576
**Socioeconomic status**	**0.13**	**0.06**	**0.039**	−0.04	0.06	0.511
Stunting (0 = not stunted, 1 = stunted)*	− 0.04	0.05	0.474	**− 0.13**	**0.06**	**0.032**
Food insecurity	0.02	0.06	0.732	−0.09	0.04	0.053
STH infection (0 = not infected, 1 = infected)*	0.05	0.03	0.127	−0.11	0.06	0.057
Grip strength	0.04	0.04	0.374	0.09	0.08	0.242
Cardiorespiratory fitness	**0.09**	**0.05**	0.049	**0.14**	**0.07**	**0.044**
**Life skills**	**Mixed multiple linear regression**					
	Boys			Girls		
Age	0.04	0.07	0.557	0.14	0.09	0.103
BMI	0.02	0.06	0.693	0.06	0.07	0.406
Socioeconomic status	**0.18**	**0.06**	**0.003**	−0.07	0.06	0.236
Stunting (0 = not stunted, 1 = stunted)*	**− 0.10**	**0.05**	**0.047**	−0.09	0.06	0.149
Food insecurity	0.02	0.07	0.817	−0.06	0.048	0.239
STH infection (0 = not infected, 1 = infected)*	− 0.03	0.03	0.387	−0.11	0.06	0.060
Grip strength	0.04	0.05	0.371	0.03	0.08	0.703
Cardiorespiratory fitness	0.05	0.05	0.363	**0.17**	**0.07**	**0.012**

Notes: School classs was used as a random intercept accross all analyses. *Being “not stunted” and being “not infected” are used as reference. *STH* Soil trasmitted helminths. Statistically significant associations are highlighted with bold font

A different picture emerged for girls (Table 2). Consistently across all academic domains, higher CRF was associated with higher grades. Furthermore, stunted growth was linked to poorer grades in language and mathematics.

### Multivariate analyses to explain inhibitory control

Table 3 provides an overview of factors that were associated with information processing and inhibitory control of attention among boys. Across all Flanker task performance indicators, age was the most important explanatory factor. Thus, higher age was associated with better information processing (higher accuracy and lower reaction time on congruent trials) and inhibitory control of attention (higher accuracy and lower reaction time on incongruent trials). Additionally, among boys, children with higher grip strength performed better in all Flanker task indices, also after excluding boys who did not reach accuracy levels higher than chance.

**Table 3:** Multiple mixed linear regression analyses, explaining Flanker task results in boys (*n* = 664)

**Flanker task: Accuracy (congruent stimuli)**	**Mixed multiple linear regression**					
	**All boys (*n*** **= 664)**			**After exclusion of boys who did not perform higher than chance (*n*** **= 612)**		
Explanatory variables	Estimate	S.E.	*p*-value	Estimate	S.E.	*p*-value
Age	**0.28**	**0.04**	**0.000**	**0.28**	**0.05**	**0.000**
BMI	−0.08	0.04	0.054	−0.06	0.05	0.267
Socioeconomic status	0.00	0.04	0.994	0.01	0.06	0.808
Stunting (0 = not stunted, 1 = stunted)*	−0.02	0.04	0.649	−0.05	0.04	0.304
Food insecurity	−0.04	0.04	0.395	0.05	0.05	0.342
STH infection (0 = not infected, 1 = infected)*	0.01	0.02	0.497	0.02	0.03	0.643
Grip strength	**0.12**	**0.04**	**0.005**	**0.08**	**0.04**	**0.033**
Cardiorespiratory fitness	0.04	0.03	0.277	0.04	0.04	0.242
**Flanker task: Accuracy (incongruent stimuli)**	**Mixed multiple linear regression**					
	**All boys (*n*** **= 664)**			**After exclusion of boys who did not perform higher than chance (*n*** **= 612)**		
Age	**0.20**	**0.06**	**0.001**	**0.29**	**0.05**	**0.000**
BMI	**−0.08**	**0.04**	**0.048**	−0.04	0.05	0.338
Socioeconomic status	0.07	0.05	0.105	0.06	0.06	0.315
Stunting (0 = not stunted, 1 = stunted)*	0.01	0.04	0.875	−0.03	0.04	0.496
Food insecurity	−0.03	0.05	0.582	−0.02	0.06	0.705
STH infection (0 = not infected, 1 = infected)*	0.05	0.03	0.117	0.05	0.04	0.197
Grip strength	**0.17**	**0.04**	**0.000**	**0.12**	**0.04**	**0.001**
Cardiorespiratory fitness	0.05	0.05	0.302	0.04	0.05	0.350
**Flanker task: Reaction time (congruent stimuli)**	**Mixed multiple linear regression**					
	**All boys (*n*** **= 664)**			**After exclusion of boys who did not perform higher than chance (*n*** **= 612)**		
Accuracy (congruent stimuli)	−0.06	0.03	0.101	−0.04	0.04	0.038
Age	**−0.47**	**0.04**	**0.000**	**−0.48**	**0.04**	**0.000**
BMI	0.03	0.03	0.215	0.05	0.04	0.141
Socioeconomic status	−0.08	0.05	0.130	−0.11	0.05	0.035
Stunting (0 = not stunted, 1 = stunted)*	0.03	0.03	0.288	0.06	0.03	0.063
Food insecurity	0.02	0.05	0.641	0.03	0.06	0.631
STH infection (0 = not infected, 1 = infected)*	0.00	0.03	0.934	−0.01	0.03	0.766
Grip strength	**−0.14**	**0.04**	**0.000**	**−0.14**	**0.03**	**0.000**
Cardiorespiratory fitness	−0.05	0.04	0.210	−0.04	0.04	0.243
**Flanker task: Reaction time (incongruent stimuli)**	**Mixed multiple linear regression**					
	**All boys (*n*** **= 664)**			**After exclusion of boys who did not perform higher than chance (*n*** **= 612)**		
Accuracy (congruent stimuli)	0.00	0.04	0.989	−0.06	0.04	0.210
Age	**−0.39**	**0.05**	**0.000**	**−0.42**	**0.04**	**0.000**
BMI	0.04	0.04	0.277	0.03	0.04	0.447
Socioeconomic status	−0.05	0.05	0.344	−0.06	0.05	0.212
Stunting (0 = not stunted, 1 = stunted)*	0.06	0.03	0.042	0.07	0.03	0.007
Food insecurity	0.00	0.06	0.954	0.02	0.07	0.810
STH infection (0 = not infected, 1 = infected)*	0.01	0.03	0.872	−0.01	0.04	0.871
Grip strength	**−0.14**	**0.04**	**0.001**	**−0.15**	**0.04**	**0.000**
Cardiorespiratory fitness	−0.07	0.04	0.088	**−0.08**	**0.04**	**0.022**

*Notes*. School class was used as a random intercept across all analyses. * Being “not stunted” and being “not infected” are used as reference. *STH* Soil-transmitted helminths. Statistically significant associations are highlighted with bold font

The results for girls are displayed in Table 4. Similar to boys, higher age was associated with better performances across all Flanker task indices. Contrary to boys, grip strength was not associated with the Flanker task outcomes in a consistent way. Importantly, our analyses show that higher CRF was associated with better information processing and inhibitory control of attention as reflected by higher accuracy/lower reaction time on congruent and incongruent trials, respectively. Finally, girls who were infected with STH had lower accuracy scores in response to congruent stimuli than non-infected girls. However, no significant differences were found in the other Flanker task domains.

**Table 4:** Multiple mixed linear regression analyses, explaining Flanker task results in girls (*n* = 613)

**Flanker task: Accuracy (congruent stimuli)**	**Mixed multiple linear regression**					
	**All girls (*n*** **= 613)**			**After exclusion of girls who did not perform higher than chance (*n*** **= 555)**		
Explanatory variables	Estimate	S.E.	*p*-value	Estimate	S.E.	*p*-value
Age	**0.28**	**0.05**	**0.000**	**0.33**	**0.05**	**0.000**
BMI	0.04	0.05	0.457	0.03	0.04	0.536
Socioeconomic status	0.08	0.06	0.186	0.06	0.05	0.181
Stunting (0 = not stunted, 1 = stunted)*	0.01	0.05	0.770	0.03	0.04	0.471
Food insecurity	0.02	0.06	0.716	0.09	0.06	0.164
STH infection (0 = not infected, 1 = infected)*	**−0.17**	**0.06**	**0.006**	**−0.17**	**0.09**	**0.047**
Grip strength	0.02	0.07	0.781	−0.03	0.07	0.656
Cardiorespiratory fitness	**0.14**	**0.04**	**0.000**	**0.12**	**0.05**	**0.013**
**Flanker task: Accuracy (incongruent stimuli)**	**Mixed multiple linear regression**					
	**All girls (*n*** **= 613)**			**After exclusion of girls who did not perform higher than chance (*n*** **= 555)**		
Age	**0.29**	**0.06**	**0.000**	**0.34**	**0.06**	**0.000**
BMI	−0.08	0.05	0.103	−0.09	0.06	0.119
Socioeconomic status	0.01	0.04	0.871	−0.01	0.05	0.848
Stunting (0 = not stunted, 1 = stunted)*	−0.08	006	0.174	−0.02	0.05	0.636
Food insecurity	−0.02	0.05	0.697	0.02	0.05	0.685
STH infection (0 = not infected, 1 = infected)*	−0.11	0.06	0.064	−0.06	0.07	0.386
Grip strength	0.01	0.04	0.874	0.00	0.06	0.984
Cardiorespiratory fitness	**0.17**	**0.04**	**0.000**	**0.13**	**0.05**	**0.007**
**Flanker task: Reaction time (congruent stimuli)**	**Mixed multiple linear regression**					
	**All girls (*n*** **= 613)**			**After exclusion of girls who did not perform higher than chance (*n*** **= 555)**		
Accuracy (congruent stimuli)	**−0.09**	**0.03**	**0.009**	**−0.09**	**0.04**	**0.021**
Age	**−0.42**	**0.05**	**0.000**	**−0.41**	**0.06**	**0.000**
BMI	−0.03	0.04	0.372	−0.03	0.04	0.426
Socioeconomic status	**0.09**	**0.04**	**0.030**	0.08	0.05	0.071
Stunting (0 = not stunted, 1 = stunted)*	0.03	0.04	0.378	0.07	0.04	0.078
Food insecurity	−0.02	0.05	0.669	−0.03	0.05	0.605
STH infection (0 = not infected, 1 = infected)*	0.03	0.04	0.370	0.02	0.03	0.572
Grip strength	−0.08	0.05	0.131	−0.09	0.056	0.104
Cardiorespiratory fitness	**−0.11**	**0.06**	**0.045**	**−0.12**	**0.06**	**0.040**
**Flanker task: Reaction time (incongruent stimuli)**	**Mixed multiple linear regression**					
	**All girls (*n*** **= 613)**			**After exclusion of girls who did not perform higher than chance (*n*** **= 555)**		
Accuracy (congruent stimuli)	−0.06	0.06	0.287	**−0.10**	**0.04**	**0.025**
Age	**−0.37**	**0.05**	**0.000**	**−0.37**	**0.05**	**0.000**
BMI	0.00	0.04	0.914	0.00	0.04	0.987
Socioeconomic status	0.03	0.05	0.529	0.07	0.05	0.191
Stunting (0 = not stunted, 1 = stunted)*	0.03	0.04	0.396	0.08	0.04	0.052
Food insecurity	−0.05	0.05	0.268	−0.06	0.05	0.219
STH infection (0 = not infected, 1 = infected)*	0.04	0.04	0.291	0.03	0.02	0.221
Grip strength	−0.07	0.05	0.148	**−0.10**	**0.05**	**0.032**
Cardiorespiratory fitness	**−0.10**	**0.05**	**0.034**	**−0.13**	**0.05**	**0.021**

*Notes*. School class was used as a random intercept across all analyses. Being “not stunted” and being “not infected” are used as reference. *STH* Soil-transmitted helminths. Statistically significant associations are highlighted with bold font

## Discussion

The present study examined multiple potential health determinants that may affect academic achievement and cognitive performance/inhibitory control of children from marginalized primary schools in South Africa. In addition, separate analyses for both sexes were conducted due to significant differences between boys and girls regarding academic achievement and cognitive performance. The key findings are that if multiple potential factors are considered simultaneously, higher family SES was most closely associated with academic achievement among boys, whereas higher levels of CRF and not being stunted explained most variance in academic achievement in girls. Higher age was associated with better performance in the Flanker task. Additionally, in boys, higher grip strength was associated with better information processing and inhibitory control of attention, whereas in girls, higher CRF levels were associated with better information processing and inhibitory control of attention.

In the present sample, 7.1% of the learners were infected with STH. This prevalence was relatively low compared to other studies with South African schoolchildren [23]. However, previous research showed that substantial differences can exist in the prevalence of STH infections even between schools located in the same geographic area [39], and that deworming programs are generally successful in keeping the infection rate low [40]. In our study, 9.5% of the 4 to 6-year old children were classified as stunted. This is similar to the percentage of stunted children reported in the 2013 first National Health and Nutrition Examination Survey [41]. Lower prevalence rates were reported by Kruger et al. [42] in grade-1 learners, with rates being similar among boys (4.1%) and girls (4.5%), but higher among students from quintile 1–3 schools (3.9–10.7%) compared to peers from quintile 4–5 schools (0.6–2.0%). The reported level of food insecurity was low to moderate in the present sample with a mean of 1.3 on a scale from 0 (food secure) to 3 (food insecure), which is in line with a previous study carried out in the same area, in which food insecurity was assessed via student reports [23].

### Associations with academic achievement

As mentioned above, the present data revealed significant sex differences with regard to academic achievement and inhibitory control. While girls had higher grades across all school subjects, boys achieved a faster reaction time with consistent accuracy in the Flanker task. Based on the standards of Cohen [43] who defined differences with 1–5.8% of explained variance as small, the sex differences found in the present study were of relatively small magnitude (2.8–3.7% of explained variance for school grades, 1.8–1.9% for reaction time). Thus, while on average girls may be achieving higher grades, the difference on the report card is likely to be not that pronounced. Nevertheless, better school performance in this age range among girls compared to boys has been frequently found in other studies [44, 45]. Potential underlying factors favouring girls are differences in motivation, effort, approaches towards schoolwork and learning styles, parental expectations and encouragement, stereotype threat, activity level, and temperament [46]. However, researchers have also emphasized the subjective side of school grades, as they depend on teachers’ perceptions and evaluations, which may lead to sex-biased treatment and/or self-fulfilling prophecies [47, 48]. While the majority of these explanations are based on samples from Europe and North-America, other factors might account for the observed sex differences in the present South African sample. For example, stunted children are more likely to miss school due to their increased infectious diseases risk [49], and to show lower academic achievements [50]. Our data further showed that in boys, children with higher SES presented with better school performances than their peers, which is in line with prior research [51]. This is plausible as higher SES might be associated with exposure to a better and more challenging learning environment at home, higher interest of parents in the education of their offspring, and more favourable educational styles [52]. By contrast, the association between academic achievement and SES was less strong among girls, maybe because in young children, girls are more intrinsically motivated at school than boys due to their perception of what are appropriate and important activities for their gender [53].

Contrary to previous studies [23, 54, 55], we did not find evidence in the multivariate analyses that STH infections are associated with academic achievement (although a tendency was observed among girls). Researchers have suggested that reduced well-being, higher fatigue, increased levels of pro-inflammatory cytokines or abdominal discomfort may explain how STH infections impact on academic achievement and cognitive function. The general low prevalence of STH infections in the present sample might have been a reason why such a relationship was not found in our study.

With regard to physical fitness, our study indicates that children with higher CRF seemed to perform better at school, which is in line with previous studies carried out in higher- and lower-income countries [56]. However, our study results also suggest that the association seems to be more pronounced in girls than in boys. This finding contrasts with previous research where physical fitness parameters were similarly associated with academic performance in boys and girls [20, 57]. However, the state of research is not entirely consistent, with some studies showing stronger associations in boys [58], whereas others found stronger relationships in girls [59]. Although speculative, it is possible that indirect pathways (e.g. mediated via self-esteem, self-discipline) may be responsible for the fact that cardiorespiratory was more strongly associated with academic performance among girls [60]. However, such indirect pathways need be tested more thoroughly in future studies. It is also possible that other (non-assessed) factors that are particularly relevant for children living in marginalized neighbourhoods are responsible for the observed results pattern. Interestingly, in the present study, no significant association was found between children’s grip strength and academic achievement although previous studies have reported significant associations between children’s muscular strength and their academic performances [56].

### Associations with inhibitory control

The fact that academic achievement is influenced by a multitude of health-related and environmental factors and not solely based on cognitive performance is reflected in the present Flanker task results, with boys performing better than girls. This was not due to a speed-accuracy trade-off, since boys had faster reaction times on both congruent and incongruent trials although accuracy did not differ between sexes. Overall, children’s sex explained less variance in inhibitory control than in academic achievement. It has been reported previously that sex differences are smaller if comparisons are based on more objective and standardized achievement tests [44]. Our findings are also in line with studies showing that boys score better in some (but not all) inhibitory control tasks [61]. Boys seem to excel in tasks which involve inhibitory control of attention (such as the Flanker task), whereas they perform similarly on tasks tapping behavioural inhibition [62]. Nevertheless, we found significant correlations between higher school grades and better cognitive performance in the computerized Flanker task independent of children’s sex. This accords well with the international literature showing that higher inhibitory control, which may reflect a more favourable executive function profile [63], translates into children’s future academic success.

As expected, higher age was associated with better performance in all Flanker task outcomes, independent of children’s sex. This common pattern reflects the functional plasticity of the developing brain [64] and highlights the substantial cognitive development that takes place during this sensitive period of life [65].

A further observation was the association of higher muscle strength with better cognitive performance in boys, whereas higher CRF was linked to better cognitive performance in girls. We are not aware of any comparable findings from the existing literature; however, previous studies have shown that children with better CRF and higher muscular strength perform better on tasks tapping inhibitory control and other aspects of executive function [66, 67]. However, few studies have found a relationship between CRF and reaction times [66, 68], whereas associations for response accuracy were more frequently observed [69–71]. Multiple physiological and psychological mechanisms have been described to explain how physical fitness contributes to better performance on cognitive tasks demanding information processing and/or inhibitory control. Building on the strength model of self-control, physical exercise targeting improvements in physical fitness demands and trains self-control resources, which in turn can be transferred into the cognitive domain by facilitating self-regulation [72]. In this model, effort is a common resource of both executive function and self-regulation that alters or maintains behaviour under specific situations. Consequently, higher self-regulation abilities with higher physical fitness may translate from one domain to the other. In addition to psychological pathways, alterations of specific cognitive processes have been suggested to account for the association of physical fitness with information processing and/or inhibitory control. A review of neurophysiological evidence from studies employing electroencephalographic measurements has found that high CRF is linked with more effective allocation of attentional resources towards the cognitive task [73]. Moreover, improved monitoring of the stimulus conflict has been found to mediate the association between this aspect of fitness and inhibitory control of attention [74]. In addition, limited evidence also suggests structural brain changes in regions associated with executive function in response to regular physical activity [75], so that the association between high physical fitness (as a result of regular engagement in physical activities) and high cognitive performance might partly be explained by such changes. Nevertheless, as highlighted by Howard et al. [76], researchers should also consider possible negative effects of physical activity on executive functions particularly among children who have little excess energy.

The significant relationship between grip strength and boys’ performances in the Flanker task is interesting. There is growing evidence in general population studies that handgrip strength is a predictor of all-cause and cardiovascular mortality [77] and mental health [78]. Studies also have shown that grip strength is closely associated with cognitive function in older people [79, 80] or patients with psychiatric disorders [81]. By contrast, research on grip strength as a health resource among children is in an early stage, and so far little evidence exists that grip strength is suitable as a predictor of children’s health [82]. To the best of our knowledge, only one study has shown that grip strength is positively associated with children’s selective attention [83], and no studies exist on the relationship between grip strength and performance in the Flanker task.

### Strengths and limitations

The present study contains strengths and limitations. Particular strengths were that we took into account the nested nature of the data because academic achievement can strongly vary as a function of school and class. Moreover, the findings are based on a relatively large sample, in which boys/girls and children from grades 1–4 are similarly represented. Furthermore, statistical software was used that is able to handle missing data in a meaningful way and a robust estimator was applied to deal with non-normally distributed data. Because sex differences existed on multiple variables, we performed separate analyses for boys and girls, and the multivariate analyses enabled us to simultaneously control for multiple explanatory factors. Importantly, independent variables were only weakly to moderately correlated with each other, and all variance inflation factors were low (VIF ≤ 1.62). Accordingly, multicollinearity was not an issue in the present study. Limitations are that the findings are based on cross-sectional data which precludes conclusions about cause and effect. Moreover, school performances depend on a multitude of factors, which were not all assessed in the present study (e.g., parental education/literacy, parental support, etc.). It should also be noted that school grades are not a fully objective measure; rather, they are dependent on perceptions and expectations of the teachers or performances of class-mates [84, 85]. While standardised measures of academic achievement are not available for this age group, in the present study, we were at least able to include an objective indicator of cognitive function that is independent from the above influences. Fourth, implementing the Flanker task with young children in marginalized settings poses some special challenges. Our impression was that some of the children (particularly the younger ones) have seen a laptop for the very first time; thus, we had to provide extensive instructions to ensure that all children have well understood the task. Moreover, all children had to perform two practice trials before the assessment started. Despite these measures, not all children reached sufficient accuracy levels. Last, we also acknowledge that generalizability of our findings is limited by the fact that all children were recruited from schools located in marginalized neighbourhoods in one peri-urban setting. We also need to be cautious with generalizing the findings to younger or older school-aged children, as the effects of CRF and grip strength might be distinct.

## Conclusions

The present study suggests that CRF and grip strength are two modifiable factors that have the potential to be improved via school-based health interventions. Further evidence is needed whether similar associations exist among children from other African countries, and whether academic/cognitive performances can be improved via structured physical activity interventions.

## Data Availability

The datasets used and analysed during the current study are available from the corresponding author on reasonable request.

## Abbreviations

ANOVA: Analysis of variance
CRF: Cardiorespiratory fitness
FIML: Full information maximum likelihood
IQ: Intelligence quotient
LMICs: Low- and middle-income countries
MCAR: Missing completely at random
MLR: Robust maximum likelihood estimator
SES: Socioeconomic status
STH: Soil-transmitted helminths
USAID: United States Agency for International Development
VO_2_max: Peak maximal oxyen uptake
WHO: World Health Organization
